# Human Metapneumovirus: Mechanisms and Molecular Targets Used by the Virus to Avoid the Immune System

**DOI:** 10.3389/fimmu.2018.02466

**Published:** 2018-10-24

**Authors:** Jorge A. Soto, Nicolás M. S. Gálvez, Felipe M. Benavente, Magdalena S. Pizarro-Ortega, Margarita K. Lay, Claudia Riedel, Susan M. Bueno, Pablo A. Gonzalez, Alexis M. Kalergis

**Affiliations:** ^1^Millennium Institute on Immunology and Immunotherapy, Departamento de Genética Molecular y Microbiología, Facultad de Ciencias Biológicas, Pontificia Universidad Católica de Chile, Santiago, Chile; ^2^Departamento de Biotecnología, Facultad de Ciencias del Mar y Recursos Biológicos, Universidad de Antofagasta, Antofagasta, Chile; ^3^Departamento de Ciencias Biológicas, Facultad de Ciencias de la Vida, Universidad Andrés Bello, Santiago, Chile; ^4^Departamento de Endocrinología, Facultad de Medicina, Pontificia Universidad Católica de Chile, Santiago, Chile

**Keywords:** human metapneumovirus, immune system, evasion, cytokines, respiratory virus

## Abstract

Human metapneumovirus (hMPV) is a respiratory virus, first reported the year 2001. Since then, it has been described as one of the main etiological agents that causes acute lower respiratory tract infections (ALRTIs), which is characterized by symptoms such as bronchiolitis, wheezing and coughing. Susceptible population to hMPV-infection includes newborn, children, elderly and immunocompromised individuals. This viral agent is a negative-sense, single-stranded RNA enveloped virus, that belongs to the *Pneumoviridae* family and *Metapneumovirus* genus. Early reports—previous to 2001—state several cases of respiratory illness without clear identification of the responsible pathogen, which could be related to hMPV. Despite the similarities of hMPV with several other viruses, such as the human respiratory syncytial virus or influenza virus, mechanisms used by hMPV to avoid the host immune system are still unclear. In fact, evidence indicates that hMPV induces a poor innate immune response, thereby affecting the adaptive immunity. Among these mechanisms, is the promotion of an anergic state in T cells, instead of an effective polarization or activation, which could be induced by low levels of cytokine secretion. Further, the evidences support the notion that hMPV interferes with several pattern recognition receptors (PRRs) and cell signaling pathways triggered by interferon-associated genes. However, these mechanisms reported in hMPV are not like the ones reported for hRSV, as the latter has two non-structural proteins that are able to inhibit these pathways. Several reports suggest that viral glycoproteins, such as G and SH, could play immune-modulator roles during infection. In this work, we discuss the state of the art regarding the mechanisms that underlie the poor immunity elicited by hMPV. Importantly, these mechanisms will be compared with those elicited by other common respiratory viruses.

## Introduction

Human Metapneumovirus (hMPV) is a respiratory virus discovered by van Den Hoogen et al. the year 2001, in samples from Dutch children with acute lower respiratory tract illness (ALRTI) (1). This virus is known to present a similar pathology and clinical symptoms as the ones reported for the human orthopneumovirus, previously known as human respiratory syncytial virus (hRSV) (2). Clinical symptoms of hMPV-infection can be manifested in both upper and lower respiratory tracts with a predominance for the latter. These symptoms are mainly associated with the appearance of coughing, bronchitis, bronchiolitis and respiratory manifestations related to the absence of airflow (2–4). Symptomatology induced by hMPV-infection is caused by a typical Th17-like immune response, characterized by the secretion of interleukin (IL)-6 and TNF-α in the lungs (5). This immune response is also accompanied by an inadequate Th2-like profile, which is characterized by the early secretion of IL-4, IL-5, IL-8, and other pro-inflammatory cytokines (6–8). Particularly, the thymic stromal lymphopoietin (TSLP), a cytokine known to impair T cell activation, promotes a delay in the Th1-like immunity and triggers the secretion of cytokines related to a Th2-like profile, resulting in high infiltration of neutrophils in the lung of infected mice (9). This abnormal response, along with an excessive mucus production by goblet cells, triggers the collapse of the respiratory airways (3, 10). Infection with hMPV in children under 2-years-old is a risk factor for the development of asthma later in life, as reported for hRSV (11, 12). It has also been reported that, in some of the most severe cases, hMPV promotes chronic obstructive pulmonary disease (COPD) and an exacerbated response, as asthma, in humans (10, 13). It has been estimated that about 10–12% of the respiratory illness in children are associated to hMPV, being considered as one of the most prevalent viruses causing hospitalization in young children (4, 14). It has also been described that hMPV is involved in hospitalizations at a rate of 1 out of 1,000 children under the age of 5 year; and 3 out of 1,000 infants under the age of 6 months. Additionally, it has been reported that prevalence of hMPV infection is equivalent to influenza virus and parainfluenza virus types 1–3 (15).

**Figure 1:**
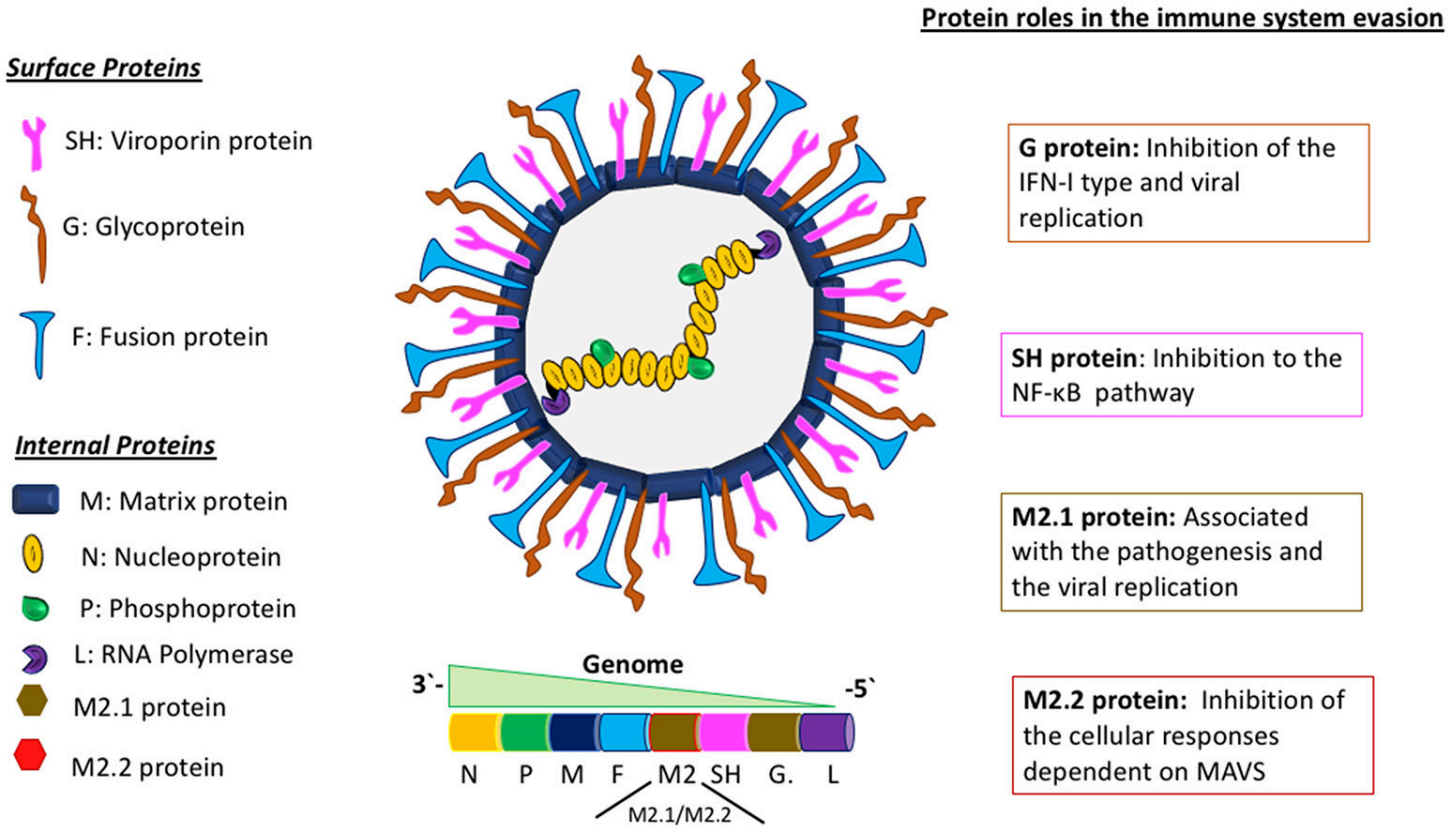
Molecular description of hMPV and the role of viral proteins in the evasion of the immune response. Schematics of the hMPV viral particle in the center and its genomic organization. In the left panel are depicted the viral proteins synthetized by hMPV, separated in two different groups: “surface proteins” and “internal proteins.” The right panel describes the main roles of some viral proteins involved in the evasion of the immune response. The proteins M2.1 and M2.2 are expressed only during the replicative viral cycle.

HMPV belongs to the *Pneumoviridae* family and *Metapneumovirus* genus. It has a negative-sense, single-stranded RNA genome of about 13 Kb of length, encoding 9 structural proteins that go as follows: 3′-N-P-M-F-M2-SH-G-L-5′ (Figure 1) (16). The different effects of these proteins on the immune system of the host are not fully characterized. The attachment G protein, one of the two proteins responsible for viral entry, has been widely studied, as it exhibits a role in the evasion of the immune response, inhibiting the interferon (IFN) pathways (17). For hRSV, this IFN pathway inhibition has been demonstrated to be caused by the non-structural proteins 1 and 2 (NS1 and NS2) (18). Remarkably, and as stated above, hMPV genome does not encode for any homolog of these NS proteins. Therefore, this shared ability to inhibit IFN pathway observed in both viruses, in the case of hMPV, is associated with the G glycoprotein, suggesting a possible gain of function for this protein, as compared with hRSV.

Despite increased incidence of hMPV infections in the last years and its impact on health care centers worldwide, neither vaccines nor effective treatments are commercially available to control or prevent infections caused by this viral agent. This is mainly due to the unclear knowledge and lack of characterization of the infection and evasion mechanisms of the immune system used by hMPV. Nonetheless, a candidate vaccine using a recombinant *Mycobacterium bovis* Bacillus Calmette-Guerin (rBCG) that expresses the P protein of hMPV has recently been developed (19). This rBCG candidate vaccine has shown promising results in mouse studies, inducing a protective adaptive immunity—both cellular and humoral—against hMPV-infection with an effective viral clearance (19–21). In this article we will discuss different evasion mechanisms developed by hMPV to prevent the generation of an adequate immune response and the role of different viral proteins involved in these virulence mechanisms.

## Persistence of HMPV in epithelial cells

It has been described that hMPV is able to persist in respiratory infected cells after the replicative viral cycle is completed. Indeed, hMPV persistence has been demonstrated in infected mice (22, 23). Measurement of plaque forming units (PFUs) from homogenized lungs of hMPV-infected mice evidenced a biphasic infection cycle, with a peak of viral particles at 7 days post-infection (dpi) and a second peak at 14 dpi. Nevertheless, the authors also reported the appearance of PFUs from the samples obtained from lungs during 28 and 60 days post-infection (22). Additionally, viral RNA was detected by Reverse-Transcription quantitative Polymerase Chain Reaction (RT-qPCR) even after 180 days post-infection. These observations suggest that hMPV may have developed mechanisms that allow its persistence inside of the host cell, resisting viral clearance until, at least, 2 months after the initial infection (22, 23).

Moreover, a study performed by Hamelin et al. found that hMPV-infected mice exhibit a strong pulmonary inflammation associated with airway obstruction (10). Further, higher viral loads were found after 5 days of infection, and these titers were still detected 12 days after, when homogenized lungs from these mice were used to infect LLC-MK2 cells *in vitro*. Similarly, detection of viral copy numbers from infected lung by RT-qPCR indicated that the highest value was detected after 5 days of the initial infection, with a considerable decrease after 21 days. Interestingly, the number of copies of the viral genetic material increased after 42 days of infection and it was still detectable until day 154. All these evidences support the idea of a chronic infection in mice. These results could suggest a relationship with the development of asthma in children infected with this virus, as chronic pulmonary disease is hallmark for the appearance of these condition that could be probably be caused by a chronic infection with this virus (11). However, further studies are require to determine if such models of infection could represent the complexity of hMPV infections in humans (10).

Despite the gaps of knowledge regarding hMPV persistence, a possible mechanism of how this virus may survive in epithelial cells for such long periods of time has been recently suggested. Typically, death of infected host cells implies a reduction in viral replication and an increase in the capacity of antigen presentation by immune cells. A study performed by Marsico et al. described in human alveolar epithelial A549 cells that hMPV is able to persist through the inhibition of the apoptosis machinery (24). This effect was associated with an increase in the expression of Bcl-2, an antiapoptotic factor from the Bcl-2 family, in the surface of hMPV-infected cells after 14 days of infection. In addition, the authors postulated a direct correlation between cellular survival and viral replication, suggesting that hMPV starts its infective cycle with a strong peak of replication and a significant increase in cellular apoptosis, followed by a decrease in viral infective rates, promoting a slow cellular proliferation, overcoming apoptosis and inducing cell cycle arrest in G2/M (24). On the other hand, hRSV-infection has been associated with an early induction of Mcl-1 -another antiapoptotic factor that belongs to the Bcl-2 family- along with more antiapoptotic factors (Bcl-W, Bcl-xL) and pro-apoptotic factors (Bid, Bax, Bak), which all could be mediated by the NF-κB pathway (25), suggesting that hMPV could also be using others factor from the Bcl-2 family.

## Role and effect of the HMPV-proteins in avoiding the immune response

Currently, the mechanisms of immune evasion used by hMPV are unclear. However, the ability of some hMPV structural proteins to inhibit some cellular pathways required to enhance viral infection have been described. Among these, the attachment or G glycoprotein has the function to recognize and promote the first interaction with the host (26). It has also been described that the G protein has the ability to inhibit the IFN-I response not only *in vitro* (17), but also in an *in vivo* model (27). The latter study showed that the G protein is associated with the recruitment of neutrophils into the alveolar space in lungs of mice infected with hMPV (27). The authors propose that this phenomenon is due to the inhibition of the IFN response, detecting important changes in molecules involved in the recruitment of neutrophils such as; CCL3, CCL4, VEGF, TNF, IL-17, and CXCL2 (27). Also, it has been demonstrated that hMPV activates the TSLP pathway and, therefore, promotes the recruitment of polymorphonuclear cells (PMNs) that secrete cytokines such as IL-13 and IL-5 (9).

A study performed by Le Nouen et al. associated a lower capacity of hMPV infected- human monocyte-derived dendritic cells (MDDCs) to present hMPV antigens to naïve T cells (28). This study described that hMPV-infected MDDCs showed lower maturation levels in flow cytometry assays. Consequently, this effect induced a poor T cell activation, as compared with MDDCs infected with a mutant hMPV that lacked both SH and G glycoprotein genes (ΔSHG). Remarkably, this effect was not observed when a mutant virus deficient in either SH or G genes was used (ΔSH or ΔG, respectively) (28). The MDDCs infected with ΔSHG virus showed an increased maturation rate, promoting the activation of naïve T cells into activated T CD4+ cells, with a Th1-like profile (28).

Interestingly, the infection rate detected in MDDCs was lower than the one identified in epithelial cells. The G protein has also been shown to induce enhanced viral replication in airway epithelial cells (AECs), but poorly in MDDCs (28). However, an *in vivo* study using the ΔG hMPV mutant virus, demonstrated that the viral replication of this mutant was impaired, promoting a small increase in the type I IFN secretion (27). Another study showed that the ΔSH hMPV mutant strain did not affect viral replication capacity when compared to the WT virus, but it did exhibit an inhibition of the NF-kB pathway in human lung epithelial cells (29). The deletion of both viral G and SH genes is related to an increase in the number of effective immunological synapses between DCs stimulated by hMPV and memory CD4+ T cells, therefore promoting the activation of these memory T cells. Altogether, it has been shown that G and SH proteins might reduce the ability of hMPV-infected DCs to activate CD4+ T cells, since both proteins are associated with decreased internalization of the virus into DCs (28). The G protein of hRSV has been described to modulate the host immune response in several ways. It has been shown that hRSV-G protein induces a biased Th2-like immune response in mice, although it can be reverted by using anti-G protein monoclonal antibodies (30). Also, hRSV-G protein can diminish the activation of DCs via interaction with DC/L-SIGN (transmembrane proteins that recognize mannose- and fucose-containing oligosaccharides), which promotes phosphorylation of ERK1 and ERK2 *in vitro* (31).

Nevertheless, some studies suggest that G protein promotes the inhibition of toll-like receptor 4 (TLR4) signaling in MDDCs, affecting the type I IFN secretion (32). The expression of type I IFN and several chemokines can be blocked by down-regulating TLR4 with siRNA, demonstrating that TLR4 is important for the activation of MDDCs induced by hMPV (32). In addition, it has been described that the G protein is also able to inhibit cellular responses induced by hMPV, by interfering with the TLR4-dependent signaling and affecting the production of cytokines and chemokines (32). Therefore, the G protein of this virus negatively modulates the immune response of the host. A study performed with TLR4-deficient mice conducted by Velayutham et al. showed that TLR4 not only plays an important role in the activation of the innate immune response to hMPV, but also contributes to disease pathogenesis (33). Inflammatory response and disease severity were seen to be diminished in hMPV-infected mice lacking TLR4, which was related to significantly lower levels of pro-inflammatory cytokines (TNF-α, IL-1β, IL-6) and chemokines in these mice. Inflammatory cells in BAL, lungs and lymph nodes were also significantly reduced in TLR4-deficient mice when compared with WT mice. Lastly, parameters like body weight loss, hyperresponsiveness and airway obstruction associated with clinical disease severity were reduced in TLR4-deficient mice (33). Similarly, immune response to hRSV induces the activation of the NF-κB pathway dependent on the expression of TLR4, however, the inflammatory response of the airway is not dependent on TLR4 (34).

The negative regulation of the type I IFN signaling pathway observed upon hMPV infection, can also be explained by the activity of the SH protein of the virus. Indeed, the deletion of hMPV SH protein is associated with an increase in the production of IL-6, IL-8 and the expression of other genes dependent of the NF-κB pathway (29). Furthermore, it has been recently determined that SH inhibits the phosphorylation of STAT1, impairing its subsequent signaling (35). Once the IFN-α receptor (IFNAR) is activated, STAT1 is needed downstream of the pathway for the expression of antiviral effector molecules (36–38). The SH protein from hRSV has been shown to prevent apoptosis in hRSV-infected cells, as this protein can inhibit TNF-α-induced death and also inhibit NF-κB activation induced by TNF-α (39). More recently, it has been proposed that hRSV-SH protein could be part of a signaling pathway that induces activation of inflammasomes, by increasing permeability and disrupting membrane architecture, which could be associated with a viral ion channel formation (40).

Although the G and SH are the most studied proteins regarding hMPV capacities to evade immunity, other proteins have also been associated to these processes. Among those, the M2-2 protein has been shown to inhibit MAVS (mitochondrial antiviral signaling), thus contributing to hMPV immune evasion, silencing the cellular responses dependent on MAVS (41). Since MAVS is needed for activation of NF-κB and IRF3, the inhibition of this protein results in enhanced viral replication and cell death, while its overexpression augments the production of IFN (activated by NF-κB and IRF3) and results in an antiviral state (42). NF-κB and IRF3 are activated by hMPV through TRAF5, TRAF6, and TRAF3, which are recruited by MAVS, thus, when suppressed by M2-2, the immune response dependent on MAVS is also inhibited, as it has been recently described (43).

Moreover, a recent *in vitro* study indicated that DCs infected with hMPV lacking the M2-2 gene produce a greater amount of cytokines, chemokines and IFNs, suggesting that the M2-2 protein has an inhibitory role on the innate immune response (44). In addition, in the same study, it was pointed out that M2-2 is capable of inhibiting the expression of genes that are dependent on MyD88 (myeloid differentiation protein response 88), an essential adapter for some TLRs, critical for the immune responses of DCs (44). hRSV strains lacking the M2-2 protein do not grow as efficiently as WT virus (45, 46). Also, it has been suggested that this protein works regulating the RNA synthesis, as infected cells with hRSV lacking M2-2 gene accumulate mRNA beyond the levels detected on cells infected with WT virus (45). The hRSV M2-2 gene has been deleted as an approach for the development of vaccine against hRSV (RSV MEDI ΔM2-2) (46).

Little is known about the role of the M2-1 protein in hMPV evasion mechanisms. However, this protein presents a critical role in hMPV pathogenesis and viral replication (47, 48). One possible characteristic suggested for the protein's role is its Zinc binding activity. Some studies suggest that mutations in this zinc binding protein can affect the infective capacity of hMPV, promoting an attenuated infection in cotton rats (47). The M2-1 protein from hRSV has a transcriptional function, as it can prevent early termination of the viral RNA transcription, which requires the formation of the RdRp complex and the interaction with RNA and the hRSV P protein. Remarkably, the hRSV M2-1 protein increases the processivity of the viral RNA polymerase (49, 50).

The matrix (M) protein is mainly associated with the assembly of the hMPV viral particle (16). It is also known that this protein promotes the production of inflammatory cytokines in MDDCs, such as IL-1β, TNF-α, IL-6, and IL-8 (4, 51). Lastly, the retinoic acid inducible gene (RIG-I) can sense hMPV infection and induce a type I IFN response, however, the hMPV-B1 strain has been described to avoid this IFN response thanks to the phosphoprotein (P), which prevents RIG-I activation (52). The M protein from hRSV is essential for viral replication and proliferation. This protein interacts with the nucleocapsids under the plasma membrane and with the envelope glycoproteins, suggesting participation in virus assembly and inhibition of transcription (53). Mice infected with hRSV lacking the M protein exhibited reduced weight loss, viral titters, and pulmonary dysfunction, as compared to control groups. Also, an important memory immune response is seen on the M-null hRSV-infected mice (54).

More studies are needed to determine the interactions between hMPV proteins and different host factors associated with the immune response, in order to understand the immune modulation during the infective process and pathogenesis, which will improve the design of efficient therapies and prophylactic approaches against this pathogen.

## Inefficient cytokine profile secretion

Several studies have been performed to characterize the immune response associated with hMPV infection—particularly, by assessing the profile of secreted cytokines (55). It has been reported that DCs obtained from mice can reduced their migratory capacity, cytokine production, and CD4+ T cell activation, when infected with hMPV, similarly as observed in hRSV infections (56–61). However, the cytokine profile induced by hMPV in humans is different from the cytokine profile induced by hRSV or influenza virus (55).

The recognition of hMPV by the innate immune system is associated with pattern recognition receptors (PRRs). This recognition in the upper and lower respiratory tract is performed by AECs, hMPV main target cells for infection, and by phagocytic cells, such as DCs and macrophages, that express PRRs and can recognize several pathogen-associated molecular patterns (PAMPs) (62). These PAMPs include double-stranded RNA and viral proteins produced during the replication cycle. The activation of PRRs renders immune cells able to promote the activation of several pro-inflammatory cytokines and chemokines (62).

Recently, it has been reported that hMPV infection induces the production of thymic stromal lymphopoietin (TSLP) as an early response to the viral infection by AECs (9). This upregulation induces an allergic-like innate immune response that drives the expression of Th1- and Th2-like cytokine such as TNF-α, IL-5, and IL-13, as a part of a late antiviral response that eventually promotes an increase in polymorphonuclear cells recruitment and an induction of mucus secretion in the mouse lung (9). In turn, a pronounced pulmonary inflammatory response and an increase in viral replication is produced upon hMPV infection.

As hRSV and hMPV exhibit similar pathologies, they have been continuously misdiagnosed among them (63). Accordingly, it has been described that the cytokine profile is similar between the two viruses, although with some differences. Indeed, Herd et al. described that the transcript levels associated with Th1-like cytokines (i.e., IL-12) and Th2-associated cytokines (i.e., IL-4 and IL-10) were significantly enhanced upon infection with either hMPV or hRSV, when compared with the respective controls in a BALB/c mouse model (64). However, levels of IL-12 were significantly higher in hMPV-infected mice when compared with hRSV-infected mice. This report also describes an increase in the secretion of IFN-γ and IFN-β (both Th1-like cytokines), along with MIP-1a and CXCL-10 (chemokines associated with the activation of granulocytes, and the chemoattraction of macrophages and T cells after the hMPV and hRSV infection) (64). Moreover, several others signaling molecules, particularly Mig, CXCL1, MIP-1β, and IP-10, are upregulated in infected mice when compared with non-infected animals.

Recently, *in vitro* studies performed by Tzannou et al., described that the immunodominant antigens of hMPV are the F, N, M2-1, M and P proteins (65). These authors also indicated that the polyclonal CD4+ T cells repertoire produced during infection was mainly associated with the secretion of IFN-γ, TNF-α, GM-CSF, and granzyme B. However, contrary to what was described above, no secretion of IL-6 or IL-10 was detected. Interestingly, mice models of hMPV-infection show that CD8+ T cells, but not CD4+ T cells nor neutralizing antibodies, are involved in the protection from reinfection. The opposite phenomena is seen in the case of hRSV-infections, where CD4+ T cells are shown to be required for protection against reinfections (66).

An increase in the secretion of IFN-γ, IL-1β, IL-2, IL-4, and IL-6 has been described in nasopharyngeal aspirates of children infected with either hMPV or hRSV that exhibit also acute respiratory tract infection symptoms; this in line with what has been previously reported in some works with mouse model (67). Remarkably, IL-2 and IL-1β secretions were higher in hMPV-infected children, when compared to those infected with hRSV. Likewise, IL-4 secretion was higher in hRSV-infected children, as compared with hMPV-infected children. These findings may imply that the robust Th2-like response that has previously been observed and extensively characterized for hRSV is not exhibited in a similar magnitude by hMPV (67). Moreover, the authors indicate that, along with this IL-1β and IL-6 increase, a Th17-like immune response associated with neutrophil-mediated asthma could be elicited during hMPV infection (67).

A study performed in Argentina using samples from newborns and children under 1-year-old, showed that low levels of IL-1β, TNF-α, IL-6, IL-8, and IL-12 were detected in samples from hMPV-infected children, as compared with children infected with hRSV or influenza virus (55). This study suggests that, despite the fact that infection caused by hMPV shares similarities with those caused by hRSV—including symptoms and pathology—hMPV induces significantly lower levels of respiratory inflammatory cytokines, as compared with the response observed in hRSV-infected children (55). Interestingly, the data obtained in this study are different to those obtained in a study performed by Jartti et al., where IL-8 levels induced by hMPV were higher than the ones induced by hRSV (68). Considering all these, it is unclear how hMPV is able to induce lung collapse in a similar way than hRSV.

It has been previously reported that many viruses are able to induce the activation of the type-I IFN pathway (i.e., IFN-α and IFN-β), promoting the proliferation and differentiation of adaptive immunity cells (69). Particularly, IFN-α and IFN-β bind to the IFNAR, prompting a signaling cascade that is mainly associated with the phosphorylation of STAT1, and its consequent dimerization with STAT2. This dimer is able to reach the nucleus and activate the transcription of several anti-viral genes that may promote viral clearance (69). *In vitro* studies have reported that hMPV is able to avoid the type-I IFN pathway, as it can modulate the first steps in the signaling cascade associated with this network. As seen for other viruses, such as influenza A virus (IAV) and hRSV, modulation of this pathway is performed by several virulence factors (69).

It has been reported for IAV that hemagglutinin, one of the surface proteins of this pathogen, is capable of inducing degradation of IFNAR, therefore inhibiting autocrine signaling associated with these molecules (69). Likewise, the NS1 protein of hRSV is able to target STAT proteins so that they are degraded through the proteasome, as described by Ramaswamy et al. (70). Particularly, for hMPV it was recently reported that the modulation of this pathway seems to be mainly through the inhibition of STAT1 phosphorylation, induced by the SH protein of this virus. The lack of phosphorylation of this protein seems to be related to a desensitization of the IFNAR to IFN-α. Moreover, other proteins have been associated with this modulation, such as G and M2.2 proteins (41, 71). Therefore, even though hMPV does seem to induce a similar response as the ones seen for other respiratory viruses, the mechanisms associated to this induction seem to be different (35, 55, 67).

Another possible reason to explain the IFN pathway inhibition are the defective interfering (DI) RNA produced during the viral replication process. It has been reported that an increase in the DI RNA can promote type-I IFN secretion in epithelial cells, where it was described that a high multiplicity of infection (MOI) of hMPV promotes an increase in the DI RNA, but a low MOI decreases the production of DI RNA and hence, an absence of type-I IFN response is observed (72). This phenomenon has only been identified in culture cell lines, such as A549 and Vero cells (72). Remarkably, this strategy to activate the type I-IFN pathway has been also described in other viruses, such as the Sendai virus (SeV), Measles virus Edmonston strain (MeVEdm), and hRSV (73).

## TLRs and their effects over the immune response induced by HMPV

TLRs are a type of receptor associated with the activation of different pathways of the immune system, mainly the innate immune system (74–78). This activation occurs by the recognition of different pathogen associated molecular patterns (PAMPs) that promote an intracellular signaling pathway in the cell. As a consequence, cytokines and chemokines are secreted in response to stimuli.

**Figure 2:**
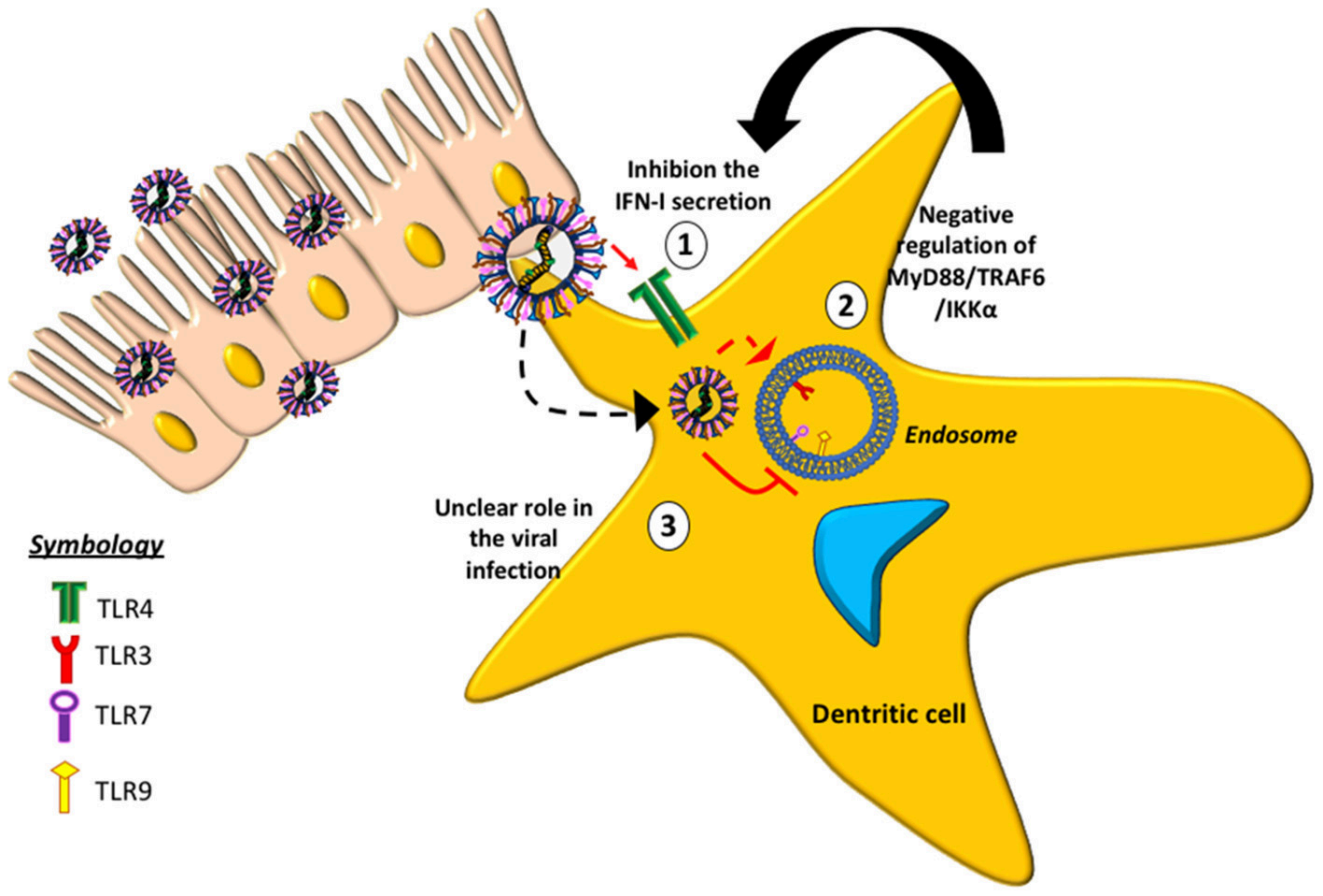
Toll-like receptors (TLRs) and their role in the evasion of the immune response induced by hMPV. The figure shows the effect of different TLRs in response to hMPV-infection. (1) At the surface of the host cell, TLR4 (green receptor) is associated with the inhibition of type-I IFN. (2) In the intracellular space, TLR3 (red receptor) is involved in the negative regulation of the MyD88/TRAF6/IKKα pathway. (3) The roles of TLRs 7 and 9 (purple and yellow receptor, respectively) in hMPV infection are currently unclear.

Some TLRs involved with hMPV-infection have been studied (Figure 2), suggesting that the absence of TLR4 in mice infected with hMPV results in a decrease in the production of cytokines, such as IFN-α/β, as well as reduced inflammatory response and disease severity (33). Similarly, a reduction in lung inflammation and disease severity has been reported in mice lacking MyD88 (79). The absence of MyD88 negatively affects the production of cytokines and chemokines and the recruitment of DCs, CD4+ and CD8+ T cells into the lungs of infected animals (79). These data indicate that both TLR4 and MyD88 are involved in the pathogenesis of the disease, the pathogenic response against the virus and in the lung inflammation.

Another role associated with MyD88 is its participation in one of the two pathways involved in the production of type-I IFN in response to *in vitro* hMPV infection, when the virus is detected by TLR3 and TLR7. Once TLR3 detects hMPV, it is able to activate IFN regulatory factor 3 (IRF3) through TRIF, whereas TLR7 activates IRF7 through MyD88 (41, 52). RIG-I and MDA5 are two helicases from the RIG-I-like receptor (RLR) family that induce signaling downstream to MAVS after detecting viral dsRNA in the cytosol (48, 56, 57). Subsequently, MAVS activates IRF3, and this in turn activates the production of type-I IFN and the up-regulation of IRF7 and NF-κB, thus producing proinflammatory cytokines and type-III IFNs (80). The absence of MDA5 in mutant mice is related with a more severe disease caused by hMPV (i.e., increased pulmonary inflammation and cellular infiltration, and a more sustained weight loss over time). This phenomenon is accompanied by an increase in viral replication and decreased production of type I-IFN, which suggests an altered antiviral response (81).

**Figure 3:**
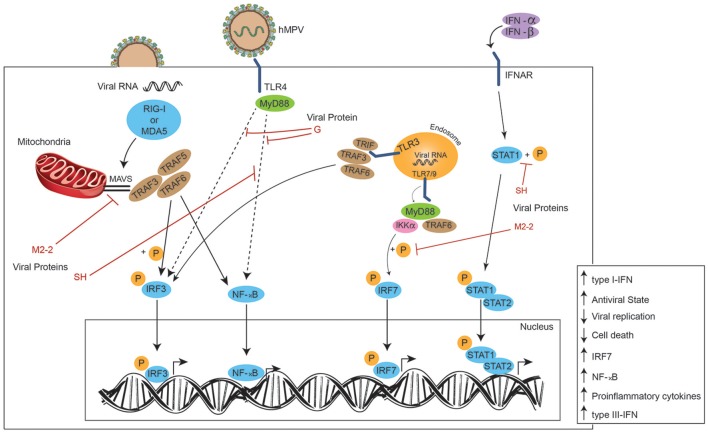
Effects of hMPV proteins on signaling pathways. Once the viral particle has fused with the plasmatic membrane, the viral RNA is delivered to the cytoplasm and recognized by MDA5 or RIG-I (PRRs), activating the IRF3 and NF-κB pathways through TRAF3, TRAF5, and TRAF6 after these factors are recruited by MAVS. After the viral proteins are translated from viral RNA inside the host cell, the M2-2 viral protein can inhibit MAVS and thus, the MAVS-dependent immune response. Viral particles are also detected by TLR4, initiating the signaling which ends in the translocation of both transcription factors IRF3 and NF-κB to the nucleus, promoting an antiviral state and the expression of type-I IFN. The G viral protein interferes with the TLR4-dependent signaling, negatively modulating the immune response of the host and promoting viral replication. The SH viral protein can inhibit the NF-κB signaling as well. Viral RNA detected inside endosomes by TLR3 and TLR7/9 results in the phosphorylation of both transcriptional factors IRF3 and IRF7, and their entry to the nucleus to promote the expression of type-III IFN and proinflammatory cytokines. The M2-2 viral protein inhibits the phosphorylation of IRF7, preventing its translocation to the nucleus. The SH viral protein induces the same effect on IFNAR signaling by preventing the phosphorylation of STAT1.

In an epithelial cell model, hMPV elicits the expression of both, RIG-I and MDA5, however, it has been suggested a more important role for RIG-I, as its inhibition results in a negative regulation of both downstream transcription factors NF-κB and IRF (Figure 3). This in turn results in a diminished production of IFN and pro-inflammatory cytokines, and an increase in viral replication, which highlights the role of RIG-I contribution to an antiviral state. This study also suggests that MDA5 seems to play a non-significant role in cellular responses induced by hMPV (80). Similarly, during early hRSV-infection (6 h post-infection), viral N protein colocalizes with RIG-I and MDA5, and later (12 h post-infection) N protein appears in close proximity to MAVS and MDA5, suggesting that the decreased IFN response -and thus the inefficient innate response observed in the experiment- is due to an interaction between the N protein and MAVS and MDA5 (82).

Once DCs have recognized the pathogen through their TLR7 and TLR9 receptors, the signaling pathways induce the activation of genes encoding for IFN-α (83–86). The M2-2 protein has also been proposed to be a potent negative regulator of the TLR7/9- dependent production of IFN-α in pDCs, since it is suggested that it is capable of inhibiting phosphorylation of Ser477-IRF7 induced by MyD88/TRAF6/IKKα (Figure 3) (87).

In addition, a study performed in neonatal mice showed that the absence of IRF3 and IRF7 is a critical factor involved in hMPV clearance. However, this response was only observed when both transcription factors were absent. It was determined through a KO mice model that the absence of IRF3 induced a decrease in IFN-β, IFN-λ2/3, and IFN-γ secretion, although IFN-α expression was not affected. Consequently, this triggered an increase in the viral loads detected in the lungs (88). Regarding IRF7, it was found that its absence decreased the expression of only four cytokines, but that viral loads in the lungs were not affected and a Th2-like profile was promoted with its characteristic increase in the eosinophil population (88). IPS-1 (IFN-β promoter stimulator 1) levels correlated positively with IFN-β secretion, and its absence resulted in an increase in IFN-a4 secretion (88). This is similar to what was observed in the IRF-7-lacking model, suggesting that IFN- α could likely be an IPS-1/IRF3-independent activation pathway of TLR7-MyD88 in plasmacytoid dendritic cells.

A recent study by Baños-Lara et al. showed a differential miRNA pattern expression in Monocyte derived DCs (MoDCs) induced by hMPV- and hRSV-infection. Therein, they report that hMPV induces a higher expression of has-miR-185–5p, a miRNA that affects different genes such as FOXO1, PDCD4 and RECK, among others; but the effect in the dysregulation of these genes is not reported (89). On the other hand, hRSV induces the expression of miR-4448, that is associated with the Akt/mTOR pathway, inhibiting apoptosis and, as a consequence, increasing cell survivor (90).

## Conclusion

hMPV is an emergent respiratory virus that each year increases its incidence and burden. Furthermore, despite its similarities with hRSV at a genomic and clinical level, the immune response triggered by both viruses are not identical. Likewise, the immune responses elicited in humans and mouse models exhibit significant differences in the cytokine secretion profile. In addition, hMPV mechanisms to escape the immune system are mostly unknown and yet to be described, although more studies regarding the understanding of this viral agent are emerging. Currently, it is known that SH and G glycoprotein are the most important proteins involved in evasion of the immune response, promoting the inhibition of an antiviral immune response triggered by type-I IFN. Also, the modulation of different intracellular TLR involved in the immune response against viral particles or intracellular pathogens is clearly a mechanism that needs to be characterized in detail. Remarkably, hamsters, mice, and even non-human primates used as animal models of hMPV-infection are, in different degrees, semi-permissive to viral infection (91), thus it is highly important to perform more studies to determine if such models are representative of an hMPV-infection in humans.

## Author contributions

JS, NG, FB, and MP-O are responsible for the writing of this review article. ML, CR, SB, and PG are responsible for reviewing the article and AK is the leading investigator and assisted in the organization and revision of the manuscript.

### Conflict of interest statement

The authors declare that the research was conducted in the absence of any commercial or financial relationships that could be construed as a potential conflict of interest.
